# High positivity values for bovine leukemia virus in human breast cancer cases from Minas Gerais, Brazil

**DOI:** 10.1371/journal.pone.0239745

**Published:** 2020-10-05

**Authors:** Emília Delarmelina, Marcelo Araújo Buzelin, Breno Samuel de Souza, Francielli Martins Souto, Juliana Marques Bicalho, Rebeca Jéssica Falcão Câmara, Cláudia Fideles Resende, Bruna Lopes Bueno, Raphael Mattoso Victor, Grazielle Cossenzo Florentino Galinari, Cristiana Buzelin Nunes, Rômulo Cerqueira Leite, Érica Azevedo Costa, Jenner Karlisson Pimenta dos Reis

**Affiliations:** 1 Laboratório de Retroviroses—Departamento de Medicina Veterinária Preventiva, Escola de Veterinária, Universidade Federal de Minas Gerais, Belo Horizonte, Minas Gerais, Brazil; 2 Instituto de Ensino e Pesquisa da Santa Casa de Belo Horizonte, Belo Horizonte, Minas Gerais, Brazil; 3 Departamento de Anatomia Patológica e Medicina Legal, Faculdade de Medicina, Universidade Federal de Minas Gerais, Belo Horizonte, Minas Gerais, Brazil; Consejo Nacional de Investigaciones Cientificas y Tecnicas, ARGENTINA

## Abstract

Bovine leukemia virus (BLV) is a retrovirus that causes lymphoma in cattle worldwide and has also been associated with breast cancer in humans. The mechanism of BLV infection in humans and its implication as a primary cause of cancer in women are not known yet. BLV infection in humans may be caused by the consumption of milk and milk-products or meat from infected animals. Breast cancer incidence rates in Brazil are high, corresponding to 29.5% a year of cancer cases among women. In 2020, an estimated 66,280 new cases of breast cancer are expected, whereas in 2018 breast cancer has led to 17,572 deaths, the highest incidence and lethality among cancers in women in this country that year. BLV infection occurrence ranges from 60 to 95% in dairy herds. In addition, there are some regions, such as the Minas Gerais State, southeastern Brazil, where the population traditionally consume unpasteurized dairy products. Taken together, this study aimed to verify if there is a higher association between breast cancer and the presence of BLV genome in breast tissue samples within this population that consumes raw milk from animals with high rates of BLV infection. A molecular study of two BLV genes was carried out in 88 breast parenchyma samples, between tumors and controls. The amplified fragment was subjected to BLV proviral sequencing and its identity was confirmed using GenBank. BLV proviral genes were amplified from tumor breast parenchyma samples and healthy tissue control samples from women, revealing a 95.9% (47/49) and 59% (23/39) positivity, respectively. Our results show the highest correlation of BLV and human breast cancer found in the world to date within the population of Minas Gerais, Brazil.

## Introduction

Breast cancer has a high incidence among Brazilian women, corresponding to 29.5% of the cancer cases in 2018 of which 25% evolve to terminal illnesses and death [1]. Each year, approximately 1.7 million new cases are diagnosed in women worldwide [2]. The greatest predisposition to the development of breast cancer are women over the age of 35, personal or family history of breast cancer, early menarche, nulliparity, late maternal age at first pregnancy, late menopause, and obesity [1]. Ongoing research for new discoveries about etiology and new treatments have brought relevant insights on this subject.

It is estimated that 10% of breast cancers are related to genetic changes including point mutations, deletions, and insertions in a set of genes that regulate cell proliferation. The most studied genes are *BRCA1* and *BRCA2*, furthermore, alterations in the *PALB2*, *PTEN*, *TP53*, *ATM*, and *CDH1* genes are considered of high risk [3]. Viral infections are also widely researched, as there is an estimate that 16% of all neoplastic diseases may have a viral origin. The oncogenic relationship is already well established between Hepatitis B virus (HBV), Hepatitis C virus (HCV) and Hepatocellular cancer, genotypes 11 and 16 of Human papillomavirus (HPV) and cervical cancer, Human T leukemia virus (HTLV) and T-cell leukemia in humans. Some viruses have been found in the human breast parenchyma such as Mouse mammary tumor virus (MMTV), Epstein-Barr virus (EBV), and Human papillomavirus (HPV) in both healthy and cancerous samples [4]. Recently, some reports have associated Bovine leukemia virus (BLV) with breast cancer in humans due to a higher prevalence of BLV-provirus in cancerous tissues compared to healthy ones [5–9]. In the USA, 59% of breast tumor tissues have been BLV positive versus 29% for control tissues [5]. In Australia, more extensively, 80% of tumors have been BLV positive while only 41% of control tissues have been BLV positive [6]. On the other hand, these data have been contested by Chinese researchers who have not found a single positive case for BLV in human breast parenchyma of 91 patients with breast tumors using RT-PCR and ELISA [10]. The experiment was contested by Buehring due to a commercial ELISA kit used to perform immunological tests in humans, considering that the test is for veterinary use only and would not be able to detect low levels of human antibodies, thus indicating the immunoblotting test as the best choice in such cases [11,12]. Buehring [12] also questioned in situ PCR methodology, which was not detailed. In Colombia, in a retrospective study, the positivity for the BLV genome in healthy tissues was markedly greater than in tumor tissues [13]. The geographic location of patients, extraction methodology, and amplification of BLV genomic material in these samples may explain the differences in the obtained results, but further investigation is still needed on this subject.

BLV is an oncogenic retrovirus that causes lymphoma in bovines and is closely related to the HTLV-1 that causes T-cell leukemia in humans. BLV infection occurs naturally in bovines, capybaras, and sheep, but *in vitro* the BLV is able to infect different cell types from a large range of species, including human cells, such as human lung embryonic cells (WI-38) and human tumor cells (ARH77 and K562) [14–16]. Neural origin human cells are also highly susceptible to BLV infection [14].

In Brazil, research on the association between BLV and human breast cancer is scarce despite the high prevalence of these two pathologies in the bovine and human population, respectively. In addition, the consumption of products from bovine origin, such as milk and meat, by the population is high compared to some regions of the globe. Recently, a study on the topic was published in the south of the country with results similar to those found by researchers from California (USA) and Australia, with a 31% positivity for BLV in tumor tissues and only a 14% BLV positivity in control tissues of healthy women [9]. This study was carried out concurrently with our work, but the patients in our study came from the state of Minas Gerais, southeastern Brazil, where the people are traditionally used to ingest raw (unpasteurized) milk and cheese and the prevalence of BLV in the dairy flock is very high, reaching more than 90% of the animals [15,16]. Thus, the present study aimed to determine if there is a higher association between breast cancer and the presence of BLV genome in breast tissue samples within this population that consumes raw milk from animals with high rates of BLV infection.

## Materials and methods

### Sampling

This study was approved by Hospital das Clinicas da UFMG and by the Research Ethics Committee of the Universidade Federal de Minas Gerais (UFMG) (protocol nº CAAE—54447716.0.0000.5149). The ethics committee waived the requirement for informed consent, since the privacy and personal identity information of all participants were protected and all the data were analyzed anonymously.

Forty-nine cases (n = 49) of invasive breast carcinomas without previous treatment (chemotherapy, radiotherapy, or hormone therapy) and thirty-nine cases (n = 39) without specific pathology and originating from reduction mammoplasty were initially considered for histopathological analysis. All samples were collected from surgical procedures performed at the Hospital das Clínicas of the UFMG and evaluated at the Mammary Pathology Medical School Laboratory of UFMG, from 2011 to 2018. Fragments of the tumors with approximately 1 cm^2^ were removed from the surgical pieces, inserted into buffered formalin 10%, and embedded in paraffin with a cold ischemia time of less than one hour.

### Deoxyribonucleic acid (DNA) extraction and Polymerase Chain Reaction (PCR)

For DNA extractions, paraffinized tissues were sectioned to a thickness of 4 μm in a microtome. To ensure the absence of contamination on the genomic material from one sample to another, some procedures were adopted, such as: using a razor for each sample, followed by cleaning the microtome with a WB40® Multi-Use oil (oil derived from petroleum) and 70% alcohol before starting a new block. A fresh pair of gloves and aluminum foil were used to collect each new sample and prevent it from coming in contact with the microtome. Next, the samples were inserted into sterile 1.5 mL microtubes and subjected to DNA extraction using the Quick-DNA ™ FFPE kit (Zymo Research) following the manufacturer's recommendations. We extracted 10 samples each time and eluted in 25 μL of elution solution, half of the amount recommended by the manufacturer to increase the amount of DNA per mL in the sample.

To verify the quality of the extracted DNA, we subjected it to amplification for the *Beta-actin* gene in 25 μL reactions (5x buffer, 37.5 mmol MgCl_2_, 10 pmol primer forward, 10 pmol primer reverse, 10 pmol dNTP, 100 U DNA polymerase enzyme from *Thermus aquaticus* (taq), 20–100 ng template) [17]. The BLV *tax* and *env* genes were amplified in two sequential nested and semi-nested PCR amplifications, respectively, in 25 μL reactions (5x buffer, 37.5 mmol MgCl2, 15 pmol primer forward, 15 pmol primer reverse, 10 pmol dNTP, 100 U taq, 20–100 ng template). All the conventional PCRs, nested and semi-nested, were performed according to the details presented in Table 1. All PCR cycles comprised 95°C for 2 minutes, followed by 35 cycles at 95°C for 30 s, TA (Table 1) for 30 s and 72°C for 30 s, finishing at 72° C for 7 minutes. The internal semi-nested forward primer of the *env* gene was designed for the present study, using Primer-BLAST Software. PCRs were performed with 20 samples with 3 types of negative controls in each run as follows: a human healthy individual PMBC control, a BLV-negative cow PBMC control (confirmed by serology and PCR) and four water controls (interspersed every 5 tubes sample). PCRs were only considered valid when no negative control amplified to the expected band size for BLV-*tax* or BLV-*env*. Two types of positive controls were used in the PCR assays as follows: a DNA from FLK cells persistently infected with BLV and a DNA from PBMC of a BLV positive cow (confirmed by serology and PCR).

**Table 1:** PCR and nested PCR parameters.

Gene	**Primers sequences**	A	AT
Beta-actin*	F: 5’GGCATCCTGACCCTGAAGTA3’	98 bp	60º C
	R: 5’CGCAGCTCGTTGTAGAAGGT3’		
BLV *tax* (extern)**	F: 5’GGCCCCACTCTCTACATGC3’	206 bp	57º C
	R: 5’AGACATGCAGTCGAGGGAAC3’		
BLV *tax* (intern, nested)**	F: 5’ATGTCACCATCGATGCCTGG3’	113 bp	57º C
	R: 5’CATCGGCGGTCCAGTTGATA3’		
BLV *env* (extern)**	F: 5’ TGATTGCGAGCCCCGATG 3’	230 bp	60º C
	R: 5’ GGAAAGTCGGGTTGAGGG 3’		
BLV *env* (intern, semi-nested)	F: 5’ CCTCCCAGGCCGATCAAG 3’	165 bp	58º C

Genes and respective primer sequences, amplicon size (A) and annealing temperature (AT).

*Described by De Souza et al., 2018 [17]

** described by Buehring et al., 2014 [8].

Analytical specificity, repeatability and reproducibility tests were performed as follows: both PCRs (BLV-*tax* and BLV-*env*) were tested with samples of PBMC (peripheral blood mononuclear cells) from individuals infected by two viruses similar to BLV, HIV (5 samples) and HTLV (5 samples) both from the family *Retroviridae*. These individuals did not have tumors or any other clinical manifestations other than those related to infection by the HIV or HTLV virus. To test the analytical repeatability both PCRs were performed by the same analyst on three consecutive days under the same conditions. To test the analytical reproducibility, both PCRs were performed by three different analysts (only once each by the second and third analyst) in different laboratories using the same protocols, but with different thermal cycler between them.

The preparation of the mix for the PCRs was carried out in a DNA-free cabinet. The pipettes used for this purpose were exclusive and never exposed to DNA. All tests were performed in duplicates. The choice of primers for amplification was made considering the peculiarities of the samples fixed in formalin and embedded in paraffin (FFPE), since these procedures are known to cause degradation of DNA [18,19]. To assess the analytical sensitivity of the PCR, DNA from the positive control (PBMCs of positive BLV cow) was used, after measuring its initial amount. Ten-fold serial dilutions were then performed, and PCR cycles of each dilution were carried out for the *tax* and *env* genes.

After the PCR, electrophoresis of the amplicons was performed in a 2% agarose gel, stained with 0.001% Ethidium Bromide, and visualized under an UV light in a transilluminator. The electrophoretic run was performed with TAE 1X buffer (40 mM Tris, 40 mM acetic acid and 1 mM Ethylenediamine tetraacetic acid—EDTA).

### DNA sequencing

To perform the nucleotide sequencing of the amplicons, the agarose gel bands were removed, purified, and concentrated using the GenElute Gel Extraction Kit (Sigma-Aldrich, USA) and the DNA Clean & Concentrator-5 Kit (Zymo Research, USA), respectively, according to the protocol established by the manufacturers. Afterward, the purified DNA was quantified using the Nanodrop Microvolume Spectrophotometers and Fluorometer equipment (ThermoFisher, USA). Nucleotide sequencing was performed using capillary electrophoresis in an ABI3730 device (Thermo Fisher, USA), using POP7 polymer and BigDye 3.1 (Thermo Fisher, USA). The sequences obtained were compared with other sequences available on GenBank using the Nucleotide BLAST tool (megablast, discontiguous megablast and blastn) to establish the degree of similarity. The *tax* and *env* nucleotide sequences obtained in this study were deposited in the same genome bank with the accession numbers MN128450-MN128470.

### Immunohistochemistry

Immunohistochemical reactions for Estrogen Receptor (ER), Progesterone Receptor (PR), Ki67 protein, and HER2 protein were performed using the non-biotinylated polymer method, following the manufacturer's protocol and recommendations (Table 2), and then evaluated according to the literature [20,21].

**Table 2:** Imunohistochemistry: Clone, manufacturer, and dilution of antibodies against HER2, RE, RP, and Ki67.

Antibody	Clone	Manufacturer	Dilution
HER2	A0485 (HercepTest™)	Dako Cytomation, United States of America	Ready to use
ER	ID5 (PharmaDX™)	Dako Cytomation, United States of America	Ready to use
PR	PGR1294 (PharmaDX™)	Dako Cytomation, United States of America	Ready to use
Ki67	MIB1	Dako Cytomation, United States of America	1:100

### Statistical analysis

Associations between BLV in breast tissue and the occurrence of neoplasia were analyzed using the Chi-square (χ^2^) test or Fisher’s exact test, where appropriate, and odds ratio (OR) with a 95% confidence interval (CI) using the Epi Info program version 7.2 (Centers for Disease Control and Prevention). For the immunohistochemistry results, a multivariate analysis on Stata 14.0 ™ (StataCorp LLC) using the logistic regression model was performed to assess the association between BLV positivity in breast tissue and prognostic markers of breast cancer (HER2, Ki67, RE, and RP).

## Results

### Sampling

The 88 samples were classified according to the histological grading criteria proposed by Elston & Ellis (1991) [20] and Page et al. (1998) [21]. Forty-nine samples were classified as breast tumors and 39 samples as healthy tissues.

### Molecular analysis

The purity of the total DNA extracted from the 88 samples ranged from 1.57 to 2.39 (260/280) and the recovery rates ranged from 12 to 617 ng/ μL. The analytical sensitivity test of the *tax* and *env* PCRs showed a detection limit of 0.08 ng of DNA for a 25 μL reaction. The amplifications of the *Beta actin* gene can be seen in S1 Fig. As can be seen in S2 and S3 Figs, no individual positive for HIV or HTLV presented amplification related to BLV-*tax* (113 bp) or BLV-*env* (165 bp) respectively. The repeatability and reproducibility tests showed 100% agreement of the results obtained by the three analysts for the two PCRs (BLV-*tax* and BLV-*env*).

**Fig 1:**
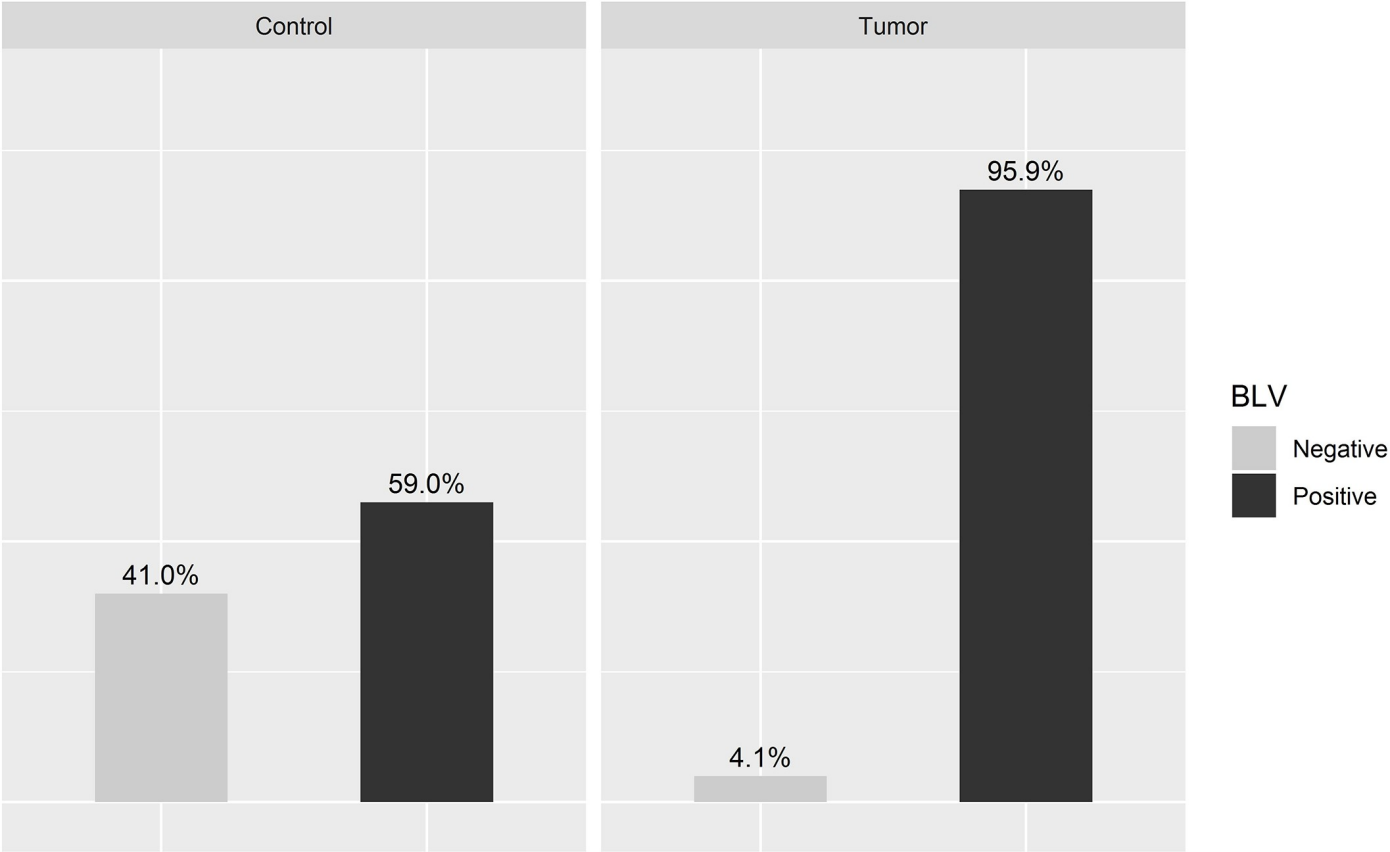
BLV genome (provirus) in malignant human breast tumors and control samples. P-value < 0.001 and odds ratio—15.8237.

Samples that amplified at least one of the viral genes were considered positive, which included 95.9% of the tumor samples (47/49) and 59% of the control samples (23/39) (Fig 1). The positivity for the *tax* gene (S2 Fig) was 93.8% (46/49) in the tumor group and 51.2% (20/39) in the control group, while for the *env* gene (S3 Fig) it was 57.1% (28/49) in the tumor group and 35.8% (14/39) in the control group.

### Sequencing

The nucleotide sequences of the BLV *tax* and BLV *env* genes obtained in this study showed varying degree of similarity ranging from 93–100% to sequences found in Genbank as follows: Colombia (MH057508.1, MH057521.1, MH057531.1, MH057530.1, MH057529.1, MH057526.1, MH041917.1, MH041915.1, and MH041913.1), Paraguay (LC080658.1 and LC080657.1), Argentina (JF288770.1, JF288769.1, and JF288763.1), Brazil (AY283061.2, and AY277947.3), Myanmar (MN128464.1), Thailand (LC102415.1), Vietnam (MH170029.1 and MH170030.1), Australia (AY700379.1), China (MF580991.1) and Russia (MN072355.1). No similarities were found with other organisms, including Human T-Cell lymphotropic viruses (HTLV-I and II), Primate T-Cell lymphotropic viruses (PTLV), and Human endogenous retrovirus K (HERV-K).

### Immunohistochemistry with prognostic markers

The individual results for the markers HER2, Ki67, Estrogen Receptor (ER), and Progesterone Receptor (PR) are shown in Table 3. According to the statistical analysis, no associations were observed between the histological variables (tumor size, HER2, Ki67, ER, and PR), and the BLV positivity in this group.

**Table 3:** Association between immunohistochemistry results with prognostic markers.

Case	Size. (cm)	Histologic type	BLV *tax*	BLV *env*	HER2	Ki67	ER	PR
1	4.00	Ductal	+	-	2	40%	+	+
2	3.00	Micropapillary	+	-	2	15%	+	+
3	3.00	Ductal	+	+	0	25%	+	+
4	3.50	Ductal	+	+	0	8%	+	+
5	3.50	Ductal	+	+	0	50%	+	-
6	4.00	Medullary	+	-	0	75%	-	-
7	3.50	Mixed	+	-	1	40%	+	+
8	2.00	Ductal	+	-	0	15%	+	+
9	6.00	Ductal	+	+	0	7%	+	+
10	2.00	Ductal, colloid	+	+	0	13%	+	+
11	2.50	Ductal	+	+	3	20%	+	+
12	3.00	Lobular	+	+	1	15%	+	+
13	1.50	Ductal	+	+	1	15%	+	+
14	1.50	Ductal	+	+	0	6%	+	-
15	3.00	Lobular	+	-	0	5%	+	+
16	5.00	Medullary	+	+	2	28%	-	-
17	3.50	Medullary	-	-	0	3%	+	+
18	2.20	Cribriform	-	-	0	19%	+	+
19	2.00	Ductal	+	+	1	50%	+	+
20	1.50	Cribriform	+	+	0	15%	+	+
21	4.00	Lobular	+	-	1	5%	+	+
22	3.00	Ductal	+	+	3	7%	+	+
23	4.50	Ductal	+	-	3	46%	+	+
24	10.00	Ductal	+	+	0	10%	+	+
25	2.50	Ductal	+	+	1	16%	+	+
26	2.50	Ductal	+	-	1	15%	+	+
27	3.50	Lobular	+	+	1	9%	+	+
28	4.00	Ductal	+	-	0	1%	+	+
29	*	Coloid	+	+	0	5%	+	+
30	4.50	Ductal	+	+	3	30%	-	-
31	1.50	Lobular	+	+	0	15%	+	+
32	5.00	Ductal	+	+	0	16%	-	-
33	1.50	Mixed	+	-	0	14%	+	+
34	400	Ductal	+	+	0	40%	+	-
35	0.60	Lobular	+	-	1	5%	+	+
36	2.50	Lobular	+	-	0	10%	*	*
37	2.50	Ductal	+	+	0	10%	+	+
38	2.50	Ductal	+	+	0	14%	+	-
39	0.50	Micropapillary	+	+	2	18%	+	+
40	1.50	Apocrine	+	-	0	25%	-	-
41	2.50	Ductal	+	-	*	*	*	*
42	3.00	Ductal	+	-	0	20%	+	+
43	1.30	Ductal	+	-	0	16%	+	+
44	4.00	Ductal	+	-	2	40%	-	-
45	2.00	Ductal	+	+	3	10%	+	+
46	3.00	Ductal	+	+	0	40%	+	+
47	1.80	Cribriform	+	+	0	12%	+	+
48	4.00	Ductal	+	-	0	17%	-	-
49	2.50	Ductal	-	+	3	32%	+	+

Individual results for size, type of tumor, positivity for *tax* and *env*, HER2 grade, proliferation marker Ki-67, estrogen receptors positivity (ER), and progesterone (PR).

* Data not available.

## Discussion

Some oncogenic viruses are related to some types of cancer in humans, such as hepatitis B and C viruses and hepatocellular carcinomas, HPV and cervical cancer, Epstein-Barr virus and Burkitt's lymphoma, HTLV -1 and T-cell lymphoma, and Herpesvirus 8 and Kaposi's sarcoma. This association may be due to a direct action of the viruses due to activation of proto oncogenes, an imbalance of cellular functions, or an indirect action by a decrease in the immune surveillance caused by immunosuppression [22].

BLV induces cancer development, predominantly malignant B-cell lymphosarcomas in its main host, the cattle. This stage of the disease is called Enzootic bovine leukosis (EBL) and it occurs only in about 5% of the infected animals [15]. Studies *in vitro* to elucidate the mechanisms of HTLV and BLV oncogenesis show that the viral protein tax appears to inhibit base excision, which repairs DNA from oxidative damage, resulting in the accumulation of mutations in the cell genome [23,24]. This can also induce mutations in *p53*, a gene that codes for a protein that regulates the cell cycle related to tumor suppression, which occurs shortly before the development of tumors, avoiding the apoptosis-inducing cascade [25], also showing that *tax* can act in conjunction with the Ha-Ras oncogene inducing cell immortalization [26]. In addition, sheep are highly susceptible to tumor formation. The latency period before the onset of the disease in sheep is shorter than in cattle and leukemia occurs usually one to four years after infection in sheep. Also, the frequency of virus-induced pathology is much higher in sheep, almost all the infected animals die within their normal lifetime, compared to only about 5% in cattle [25]. In other animals, the susceptibility of BLV-induced cancer is little known. In humans BLV has been associated with breast cancer, but the results are still contradictory. Some authors have failed to find evidence of this relationship [10,13], while others claim that such an association exists [5–9]. Our results show a high occurrence of the BLV (provirus) genome in tumor mammary parenchyma samples: 95.9% (47/49), against 59% (23/39) in healthy control tissues, demonstrating a high association of BLV with human breast cancer in the studied population, corroborating most of the results published. The data were well supported by statistical analysis as seen in Fig 1 (P-value < 0.001 and odds ratio—15.8237). The precautions taken during DNA extraction, PCR runs and nucleotide sequencing procedures, besides the high similarity with BLV sequences found in GenBank, guarantee the reliability of the results obtained in this study. In the case of PCR for BLV-*env*, some non-specific amplicons can be seen on the gel (S3 Fig) and we think that it was due to the origin of the samples (PBMC DNA), which were far better preserved than samples of formalin-fixed and paraffinized tissues, such as the samples of breast tissue used in this work.

Other gene regions such as *gag*, *pol*, and *env* are often eliminated from cellular DNA as the malignancy of the tumor progresses, which happens less frequently with the *tax* region [27]. *Tax* is still the most important gene for viral infectious potential [25]. Thus, in addition to the *tax* region being used as an amplification target, the use of nested-PCR combined with the amplification of small fragments increased the accuracy of the tests, supported by the high analytical sensitivity. Buehring and collaborators [8] used primers that generate larger DNA fragments than those in this study, which could lead to an underestimation of the positivity of the samples analyzed for the BLV. Another similar study carried out in southern Brazil showed a lower positivity index, a 30.5% positivity for BLV in tumor tissues and a 13.9% BLV positivity in control tissues [9]. This study used the same protocol described by Buehring and collaborators [8], which could be a possible explanation for the considerable difference between the results found in the South of Brazil and our results in Minas Gerais State. In our view, the use of primers for the highly conserved viral gene region, the *tax*, together with the amplification of small fragments in cases of FFPE tissues increased the chances of BLV being detected in this study. BLV serology in humans could not be performed, because commercial kits are for veterinary use only, so there are no specific kits to test humans. Moreover, this study was retrospective and blood samples were not available.

For many years, it was believed that the human species were not susceptible to BLV, but due to the high ratio of consumption of bovine products, in addition to a close contact between humans and cattle in various human cultures, research on this subject has been increasing. The interest of this study of searching the presence of BLV in human tissues in the State of Minas Gerais—Brazil stems from the high occurrence of the virus in the dairy cattle flock in that state, exceeding 90%, combined with a tradition of consuming raw milk and fresh products such as an artisanal cheese called “Queijo Minas Artesanal”. This cheese is made from unpasteurized raw milk, which provides the identity that characterizes artisanal cheese from Minas Gerais [28]. In addition, as in other parts of the world, breast cancer is an important public health problem in this population with a prevalence of 30% among all cancers and mortality rates of 25% [2]. In fact, the high association between BLV and breast cancer in humans found by Buehring et al (2017) [6] in Australia may be due to the same cause seen in the population of this study, since Australians are high consumers of bovine milk and its products. Furthermore it cannot be exclude the possibility of other factors associated with cancer induction and tumor development in some BLV-infected individuals and not in others such as: time of infection, presence of co-infections, individual differences in life habits.

Other possible forms of human infection have been attributed to close contact between humans and bovines since their domestication, consumption of raw or undercooked meat, production of vaccines from cell cultures that use fetal bovine and the direct contact with contaminated meat from butchers and slaughterhouses. However, all these forms of contamination are yet to be confirmed.

The results obtained in this study showed that there is no correlation between the cancer prognosis markers RE, RP, HER2, and Ki67 used in the laboratory routine for monitoring and treating patients, with the presence of BLV in tissues. However, the small number of tumor tissues negative for BLV (n = 2) did not generate good statistical support for this type of analysis. Other studies that analyzed this correlation did not find significant values [7,9]. This suggests that BLV is not related to the prognosis of the disease and consequent patient survival.

Our results confirm the presence of BLV genome in breast tissues of women in the State of Minas Gerais and show a statistically significant positive association between virus infection and breast cancer in this population. Previous studies indicate that this infection may be related to the ingestion of contaminated dairy products, however, a limiting factor of this study was not having access to patients' clinical data with information on places of residence and eating habits such as the consumption of milk and dairy products from bovine origin. Therefore, further studies are needed to elucidate the viability of BLV in these substrates and the route of transmission to humans, as well as to determine the role of BLV in the development of human malignant neoplasms, so that preventive measures can be taken.

## Conclusions

The results of the present study have shown the highest association to date between BLV and human breast tumors in the Minas Gerais State, Brazil. Such correlation could be attributed to the high prevalence of BLV in the region’s cattle associated with the high consumption of milk and dairy products without adequate thermal treatment by this population. Studies to verify the viability of BLV in milk and storage of dairy products in different conditions with subsequent *in vitro* and *in vivo* infectivity tests are necessary to ensure that this route of infection can be possible.

## Supporting information

S1 Fig *Beta-actin* amplification in agarose gel. (TIF)

S2 Fig BLV-*tax* amplification in agarose gel. (TIF)

S3 Fig BLV-*env* amplification in agarose gel. (TIF)
