# Supplementary figures and images for: High positivity values for bovine leukemia virus in human breast cancer cases from Minas Gerais, Brazil
Source: PLoS One. 2020 Oct 5;15(10):e0239745. doi: 10.1371/journal.pone.0239745 (PMC7535047; doi:10.1371/journal.pone.0239745)

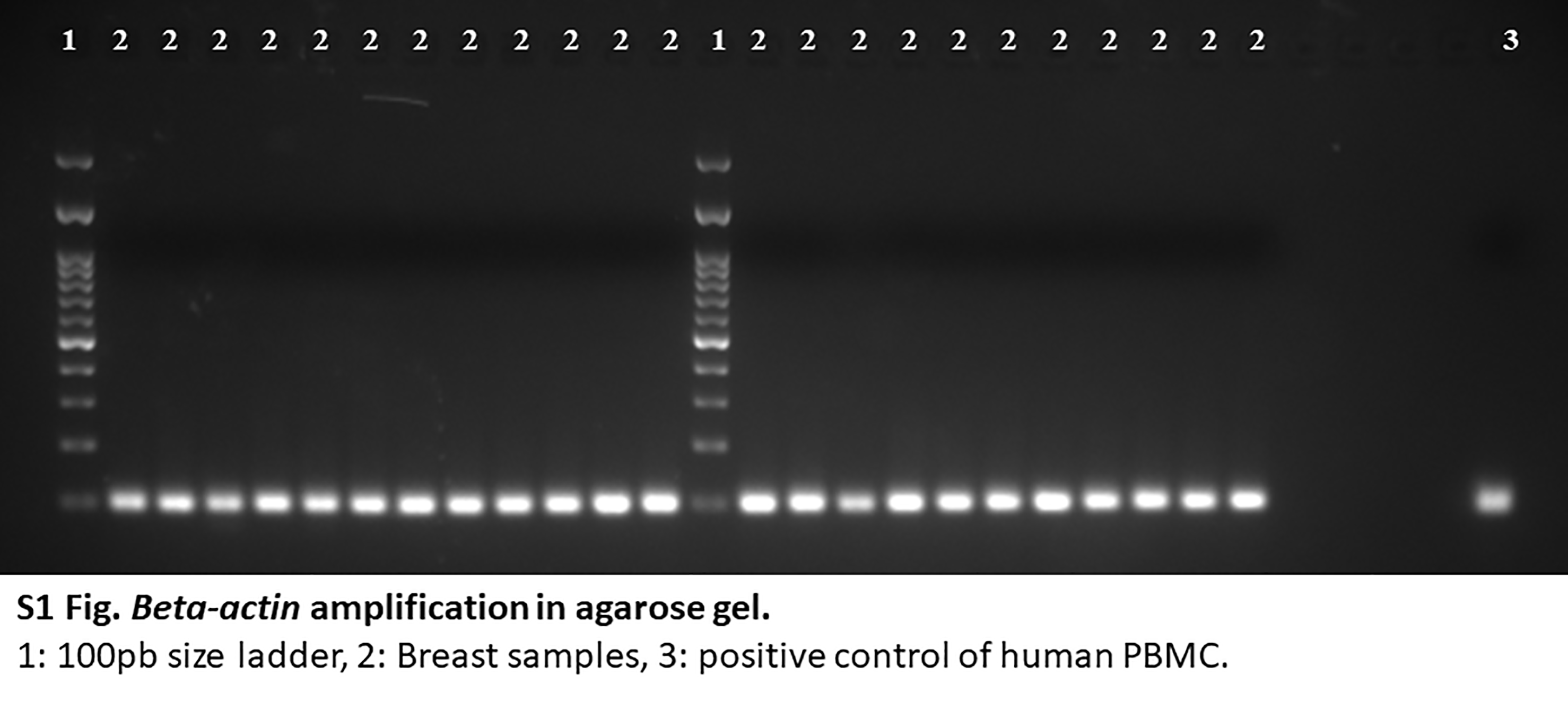

Supplement: S1 Fig — (TIF) [file pone.0239745.s001.tif]

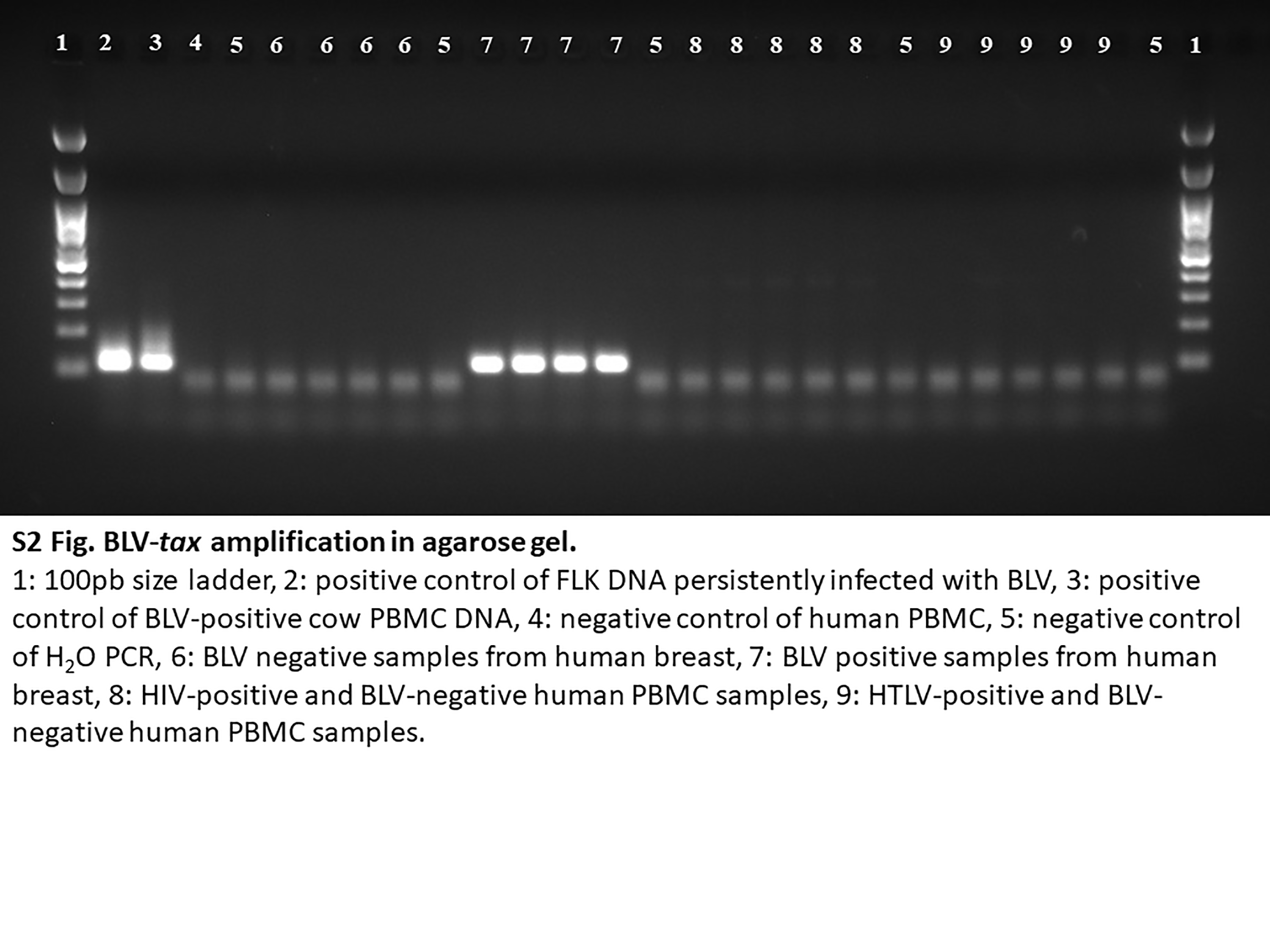

Supplement: S2 Fig — (TIF) [file pone.0239745.s002.tif]

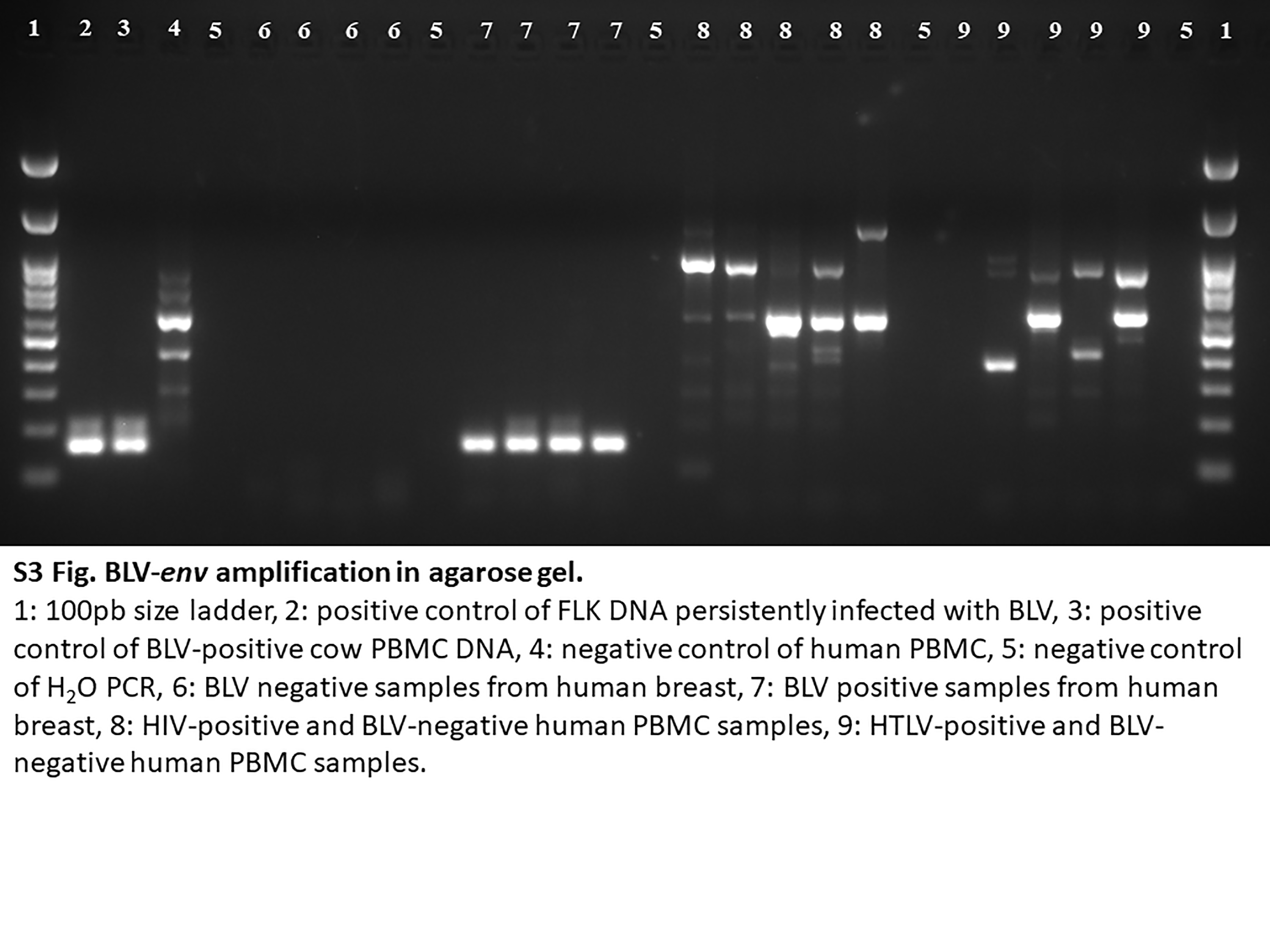

Supplement: S3 Fig — (TIF) [file pone.0239745.s003.tif]
